# A Comparison of the Behavioral and Psychological Symptoms of Dementia (BPSD) in Early-Onset and Late-Onset Alzheimer’s Disease - A Study from South East Asia (Kashmir, India)

**DOI:** 10.7759/cureus.625

**Published:** 2016-05-27

**Authors:** Raheel Mushtaq, Charles Pinto, Shah Faisal Ahmad Tarfarosh, Arshad Hussain, Sheikh Shoib, Tabindah Shah, Sahil Shah, Mushbiq Manzoor, Mudassir Bhat, Tasleem Arif

**Affiliations:** 1 Memory Clinic and Geriatric Clinic, Postgraduate Department of Psychiatry, Government Medical College, Srinagar, J & K, India; 2 Department of Psychiatry, TN Medical College and BYL Nair Hospital, Mumbai; 3 MBBS, Acharya Shri Chander College of Medical Sciences and Hospital, Jammu, J & K, India; 4 Department of Psychiatry, Government Medical College, Srinagar, J & K, India; 5 MBBS, Government Medical College, Srinagar, J & K, India; 6 MBBS, SKIMS Medical College, Srinagar, J & K, India; 7 Postgraduate Department of Radiology, Government Medical College, Srinagar, J & K, India; 8 Medical Officer, Government Health Services, Kashmir, J & K, India

**Keywords:** alzheimer disease, dementia, bpsd, nbd, memory, geriatric neurology, neuropsychiatry, psychology

## Abstract

**Background:**

A gradual increase in the longevity due to advancement of treatment modalities and a subsequent surge in elderly population in India have led to a growing curiosity in the geriatric age group with Alzheimer’s disease (AD). Behavioral and psychological symptoms of dementia (BPSD) represent epiphenomena of AD. However, no comprehensive study has been carried out in South East Asia (Kashmir, India), to assess the behavioral and psychological symptoms in subtypes of AD.

**Objectives:**

The purpose of this study was to assess BPSD in early-onset Alzheimer’s disease (EOAD) and late-onset Alzheimer’s disease (LOAD).

**Material and Methods:**

The study was conducted in the Memory clinic of the postgraduate department of psychiatry, Government Medical College, Kashmir, India from January 2012 to March 2014. The diagnosis of AD patients was done according to NINCDS-ADRDA criteria. A total of 80 patients of AD were screened (40 with age of onset less than 65, and 40 with age of onset greater than 64). Neuropsychiatric inventory (NPI) was the instrument used for evaluating symptoms of BPSD. The data was analyzed using paired t-test.

**Results:**

The mean age of presentation of EOAD and LOAD was 63.10 years and 84.28 years, respectively, and the difference between the two was found to be statistically significant. The LOAD group had significantly higher symptom severity for delusions, agitation, anxiety, disinhibition, and nighttime behavioral disturbances (NBD) than the EOAD group (p ≤.0001).

**Conclusion:**

The behavioral and psychological symptoms are significantly severe in late onset subtype compared to the early onset subtype of Alzheimer’s disease in the Kashmiri (Indian) population.

## Introduction

Alzheimer’s disease (AD) is the most common type of dementia and is a worldwide health problem, especially in a developing country like India [1-3]. AD is a neuropsychiatric disorder characterized by the presence of cerebral atrophy, extracellular amyloid plaques, and intraneuronal neurofibrillary tangles [4-7]. The condition affects 5% of the population aged over 65 years and more than 20% of the population over 85 years. Although AD is considered a progressive cognitive disorder, various non-cognitive symptoms accompany all stages of this disease. The incidence of AD has increased over the years from 4.5 million in 2000 to 5.3 million patients in 2008 in the United States of America. The incidence of AD is expected to exceed 13.8 million by 2050 [2, 5-6, 8]. The frequency of AD in India varies from 0.34% to 1.5% above 60 years of age [9]. A review of the literature shows that two types of AD have been described--early-onset and late-onset Alzheimer’s disease. AD that occurs before the age of 65 years is called as early-onset Alzheimer’s disease (EOAD). It is considered to have an aggressive course with relatively shorter survival time. AD that occurs after the age of 65 years is called as late-onset Alzheimer’s disease (LOAD) [4]. It is not known whether EOAD and LOAD are variants of the same disease or if these are two distinct entities. Although neuropathological findings are seemingly the same in both types with phenotypic differences existing between them, considering the age of onset is an important determinant of the diversity observed in the disease [1, 5].

AD is characterized by loss of cognitive and noncognitive functions in various domains. The loss of these functions causes significant disability in activities of daily life. The appearance of noncognitive symptoms occasionally occurs before the onset of cognitive symptoms. Noncognitive symptoms include behavioral and psychological symptoms of dementia (BPSD) and neurological symptoms. The mechanism by which BPSD occurs in some patients with AD is not completely understood [4]. Various studies have shown that the origin of BPSD in AD is not only due to psychological and genetic factors, but anatomical and biochemical abnormalities are also responsible for it. The psychological factors that are implicated in the origin of BPSD include pre-morbid neuroticism and low frustration tolerance [8]. Serotonin transporter promoter gene, serotonin receptor 2a gene (5HT receptor 2a), and dopamine receptors genes (DRD1 allele B1 and DRD3 allele) are found to be associated with BPSD in AD [3-4]. Psychotic symptoms like delusional misidentification have been found to be associated with low neuron count in the hippocampus and dorsal raphe. Pathological changes in the cholinergic system through denervation of the frontal and temporal cortices, modifications in the adrenergic and serotonergic systems and greater density of NFT (Neurofibrillary tangles) in the neo-cortex are also found to be associated with BPSD in AD. Further, decrease in metabolism of prefrontal, left frontal temporal, and right parietal cortices are also found to be associated with BPSD [3-4, 6, 8].

Various standardized instruments like Neuropsychiatric Inventory (NPI), behavior pathology in Alzheimer's disease (BEHAVE-AD) scale and Alzheimer's disease assessment scale (ADAS) are used to assess BPSD in AD [5]. Early detection of BPSD is extremely important because these symptoms not only induce noticeable disability in demented patients, but also increase caregivers' stress. BPSD in AD patients increase impairment in daily living activities, accelerate cognitive decline, and worsen the quality of life of patients [5]. The incidence of BPSD in AD ranges from 61% to 92% [5]. Since the frequency of BPSD is highly variable, the correct assessment of these symptoms may contribute to a better diagnosis and treatment, and could be a powerful tool to evaluate the efficacy of any therapy directed toward the improvement of behavioral disturbances. A large number of studies have been conducted on AD in western countries [1-5]. However, no comprehensive study about this common condition has been carried out in India. The few published studies are sporadic and generally focus on its epidemiology [10-12]. In view of the above, a comprehensive study to investigate BPSD in EOAD and LOAD in the Kashmiri (Indian) population was carried out.

## Materials and methods

### Setting

The study was conducted in the Memory Clinic of the Postgraduate Department of Psychiatry, Government Medical College, Srinagar, Kashmir, India from January 2012 to March 2014. The Government Medical College and its associate hospitals provide care to the whole of the Kashmir region, along with the adjoining areas of the Jammu and Ladakh regions (population of over 6 million).

### Subjects

A total of 80 patients with AD were screened (40 with age of onset less than 65 years, and 40 with age of onset greater than 64 years). The two groups were divided by the conventional division line of the 65 years. Each patient had a structured clinical interview, laboratory routine exams, physical and neurological examination, and imaging (CT or MRI).

### Alzheimer’s disease diagnoses

The diagnoses of AD were done according to NINCDS-ADRDA criteria [13] by two experienced psychiatrists having at least two years of experience in our Memory Clinic.

### Inclusion criteria

1. Patients with a one-year history of unchanged symptoms after the onset of BPSD. This was done as BPSD fluctuates over time and can subside for long periods [14].

2. Diagnosis of probable AD according to the NINCDS-ADRDA criteria [13].

3. Availability of a reliable caregiver. A reliable caregiver was defined as someone who was able to ensure the patient’s compliance with assessment procedures and who contacted the patient at least twice weekly, with one contact being a personal visit.

4. Classification in mild to moderate severity using the mini-mental state examination (MMSE).

### Exclusion criteria

1. Patients with a MMSE score below 10.

2. Patients with a past history of psychiatric illness and/or any neurological illness that could interfere with neuropsychological tests.

### Instruments

The instruments used for BPSD evaluation were applied at the time of the diagnosis by two trained psychiatrists. The instruments used in assessing behavioral and psychological symptoms in subtypes of AD included:

1. Mini-Mental State Examination (MMSE)

It is a brief 30-point questionnaire test that is used to estimate the severity of cognitive impairment [15].The instrument had to be culturally and linguistically acceptable to the local Kashmiri-speaking population. Hence the Kashmiri version of MMSE, developed by Raina SK, et al., was used [16]. In the Kashmiri version of MMSE, the selected items from the English version of the MMSE have been translated into Kashmiri and also back translated into English. It has good reliability and validity [16].

2. The Clinical Dementia Rating (CDR)

It is a scale with good reliability and validity used to assess and quantify the severity of symptoms of dementia (i.e., its 'stage') [17].

3. The Neuropsychiatric Inventory (NPI)

It is an informant rating scale used to measures various behavioral symptoms in AD like delusions, hallucinations, agitation/aggression, dysphoria/depression, anxiety, euphoria/elation, apathy/indifference, disinhibition, irritability/lability, aberrant motor behaviors (AMB), nighttime behavioral disturbances, and appetite/eating disturbances. An informant rates the frequency and severity of each of these dimensions; the final score is obtained by multiplying the first two scores. In NPI, every domain has a screening question. If it is not answered positively, the interviewer moves to the next domain. If it is answered positively, then specific behavioral symptoms are assessed within that domain. If any of these symptoms are presented, they are rated on a 4-point frequency scale and a separate 3-point severity scale. The score for each dimension ranges from 0 to 12. The maximum total score in the 12-item version of NPI is 144 [18].

### Consent and approval

The study was done after obtaining clearance from the ethical committee of the Government Medical College, Srinagar, India, and no grant was funded by the committee. The patients gave their consent before being subjected to various tests.

## Results

The study sample consisted of 80 patients: 40 with EOAD, and 40 with LOAD. Both groups were matched for education, gender, MMSE, disease duration, and severity. The mean age of presentation of EOAD and LOAD was 63.10 years and 84.28 years, respectively, and the difference between the two was found to be statistically significant. The significant mean differences were found for age of onset and MMSE (p <0.05). Table 1 shows the age of onset, presentation, mean MMSE, and education in our patients with EOAD and LOAD.

**Table 1:** Age of onset, presentation, and mean MMSE and Education in EOAD and LOAD Note: Group mean and standard deviation (SD) in years t = T score C.I. = (Confidence interval) MMSE = Mini Mental State Examination

	EOAD Mean (S.D.)	LOAD Mean (S.D.)	t	p-value	95% C.I.
Age at presentation	63.10 (1.12)	84.28 (2.17)	-54.72	≤.0001	-21.94 to -20.40
Age of onset	60.60 (1.15)	78.80 (1.95)	-50.83	≤.0001	-18.93 to -17.48
MMSE	19.98 (0.89)	18.15 (.770)	9.8	≤.0001	1.45 to 2.19
Education	10.78 (0.92)	10.50 (0.84)	1.39	0.168	-0.11 to -0.66

With regard to the NPI scores, the LOAD group had significantly higher symptom severity for delusions, agitation, anxiety, disinhibition, and nighttime behavioral disturbances than the EOAD group, and the differences were found to be statistically significant (p <0.05). Table 2 shows BPSD in our patients with EOAD and LOAD.

**Table 2:** BPSD in early-onset Alzheimer’s disease (EOAD) and late-onset Alzheimer’s disease (LOAD) Note: Group mean and standard deviation (SD) in years t = T score C.I. = (Confidence interval) NPI = (Neuropsychiatric Inventory)

	EOAD Mean (S.D.)	LOAD Mean (S.D.)	t	p-value	95% C.I
NPI Delusions	0.97 (0.42)	1.37 (.49)	-3.90	≤.0001	-0.603 to -0.196
NPI Hallucinations	0.30 (0.46)	0.47 (0.50)	-1.61	0.11	-0.391 to .041
NPI Agitation	1.20 (0.40)	2.00 (0.00)	-12.49	≤.0001	-0.927 to -0.672
NPI Depression	2.57 (0.81)	2.5750 (0.74)	.00	1.000	-0.347 to .34758
NPI Anxiety	2.57 (0.50)	3.0250 (0.91)	-2.71	0.008	-.77957 to -0.120
NPI Euphoria	0.45 (0.50)	.4500 (0.50)	0.00	1.000	-0.224 to 0.224
NPI Apathy	3.32 (0.79)	3.4500 (0.71)	-0.73	0.462	-0.461 to 0.211
NPI Disinhibition	0.12 (0.33)	1.0000 (0.00)	-16.52	≤.0001	-0.980 to -0.769
NPI Irritability	1.97 (0.15)	2.0000 (0.00)	-1.00	0.320	-0.074 to 0.024
NPI Aberrant Motor Behavior	1.00 (0.18)	1.78 (0.188)	-18.80	≤.0001	-0.862 to -0.697
NPI Nighttime Behavioral Disturbances	1.35 (0.48)	2.47 (0.71)	-8.24	≤.0001	-1.396 to -0.853
NPI Eating Disturbances	1.00 (.00)	1.25 (0.43)	-3.60	.001	-0.388 to -0.111
NPI total	17.37 (1.21)	21.70 (1.30)	-15.35	≤.0001	-4.885 to 3.764

The data was analyzed using paired t-test, and the mean difference between the two groups was considered to be statistically significant (p ≤0.05). Demographic quantitative characteristics were presented as mean and standard deviation (SD).

## Discussion

The burden of dementia in India, particularly of AD, is increasing due to increase in life expectancy. There has been growing importance of BPSD in AD over the last few years, particularly with regard to the quality of life of patients. BPSD is a term used to delineate diverse range of psychological responses, psychiatric symptoms, and behaviors occurring in any type of dementia including AD [19]. The average onset of BPSD in AD is 45-50 months after diagnosing AD. The delay in the onset of BPSD in AD may suggest that a significant and long-lasting period of neurodegeneration is required for BPSD to appear [20-21]. BPSD represents an important clinical dimension of AD that has been ignored from both research and therapeutic points of view in India [19].

Two arbitrary types of AD have been described, i.e., EOAD and LOAD [5]. Earlier, EOAD and LOAD were considered totally distinct from each other. However, the distinction vanished when similar neuropathology was found in both the subtypes [21]. Studies have found that EOAD is distinguished from LOAD by increased parietal atrophy and lesser hippocampal atrophy [22-23]. The findings of this study revealed that LOAD had more deterioration in MMSE scores compared to EOAD. This finding of the study is contrary to other studies. Most of the earlier studies report no significant difference in the MMSE scores of both the groups. However, these studies report more functional deterioration in LOAD compared to EOAD [4]. The finding of the study can be explained by the fact that average age of LOAD patients was 84 years, twenty years older than EOAD (64 years). Increased age is associated with fronto-subcortical atrophy and decreased speed of information processing, which could possibly interfere with impaired MMSE scores in LOAD patients..

The study also showed that the LOAD group had significantly higher symptom severity for delusions, agitation, anxiety, disinhibition, and nighttimes behavioral disturbances compared to EOAD group. This finding of the study is consistent with other studies [21]. In a study by Spalletta G, et al. (2013), he found higher symptom severity of anxiety (NPI Anxiety), disinhibition (NPI Disinhibition), night time behavioral disturbance (NPI Behavioral Disturbance), delusions (NPI Delusion), agitation (NPI Agitation), and total NPI scores in LOAD compared to EOAD. However, in our study in addition to the above symptoms, apathy as well as motor behavior (NPI Aberrant motor behavior) was found to be increased. The possible reason for this could be that as the disease progresses, delusions and agitation become more prominent. On the other hand, apathy is the most persistent and frequent NPS throughout all stages of AD. Further, limited review of literature shows the role of various factors like some population being more prone to exhibit certain NPS related to their genetics, medical co-morbidities, lifestyle choices, brain region atrophy, or involvement of neurotransmitters [21].

Both types of AD (EOAD and LOAD) in the study were associated with psychotic features including delusions and hallucinations. This is in accordance with the other studies. However, delusions are known to occur more frequently compared to hallucinations in LOAD [4, 20-21]. With regard to the NPI scores, the LOAD group had significantly higher symptom severity for delusion compared to the EOAD group [p ≤.0001]. The types of delusions that occur in AD are persecutory delusions and misidentification phenomena. The NPI scale used in characterization of delusions in patients of AD in the study involves both persecutory delusions as well as misidentification syndrome [21]. Moreover, it is seen that delusions and irritability are symptoms that vary substantiality with the degree of cognitive decline. Preferably, this might also be the reason why LOAD patients with impaired cognitive decline (decreased MMSE) have more severity of delusions compared to EOAD patients, thus, correlating the relationship of cognitive symptoms in AD with noncognitive symptoms, i.e., BPSD (delusions) [20-21].

It is a quite well known fact that AD patients have increased verbal and physical aggression, and thus, the handling of the patients by their caretakers is quite difficult. NPI scale underlines resistance to somatic and verbal concern [18, 24]. The finding of the study revealed that the NPI scores for symptom severity of agitation in LOAD group was significantly higher, compared to the EOAD group, and the difference was found to be statistically significant (p ≤.0001). Several other studies have found similar results [20-21]. Several authors agree that anxiety is often associated with other neuropsychiatric symptoms such as depression, psychosis, aberrant motor behaviour, disinhibition, euphoria, irritability, and agitation [25-27]. In the study, a higher severity in anxiety score was also associated with severity in psychosis, aberrant motor behavior, disinhibition, irritability, and agitation.

Indian culture and societal attitudes necessitate caring for the elderly in the home environment rather than nursing homes. The majority of the Indian population lives below the poverty line, and the management of BPSD with compromised cerebral functions is rather difficult [28]. Low-cost interventions like providing information about BPSD and educating the family members about the disease can be effective in the Indian settings. BPSD is generally considered to be more amenable to interventions than the cognitive symptoms of dementia [28]. Thus, early detection of BPSD is extremely important because these symptoms not only induce noticeable disability in demented patients, but also increase caregiver stress. Studies on BPSD in subtypes of AD appear to be least understudied and least explored in India. Further studies should be done in large samples on subtypes of AD assessing BPSD epiphenomenology thoroughly.

### Limitations

1. The sample size was small; therefore the generalization of the results may be questioned.

2. Another limitation included the possibility of selection bias as the sample was drawn from one hospital only.

## Conclusions

Although research on the subtypes of AD appears to be understudied and least explored in India, our study was a maiden attempt to understand the epiphenomenology of cognitive symptoms in subtypes of AD. Wider dysfunctions in BPSD, especially higher symptom severity for delusions, agitation, anxiety, disinhibition, and nighttime behavioral disturbances, were observed in LOAD compared to EOAD in the Kashmiri (Indian) population. 
